# Usefulness of IVD Kits for the Assessment of SARS-CoV-2 Antibodies to Evaluate the Humoral Response to Vaccination

**DOI:** 10.3390/vaccines9080840

**Published:** 2021-07-31

**Authors:** Krzysztof Lukaszuk, Jolanta Kiewisz, Karolina Rozanska, Malgorzata Dabrowska, Amira Podolak, Grzegorz Jakiel, Izabela Woclawek-Potocka, Aron Lukaszuk, Lukasz Rabalski

**Affiliations:** 1 Invicta Research and Development Center, 81-740 Sopot, Poland; krzysztof.lukaszuk@invicta.pl (K.L.); karolina.rozanska@invicta.pl (K.R.); amira.podolak@invicta.pl (A.P.); 2 Department of Obstetrics and Gynecology Nursing, Medical University of Gdansk, 80-210 Gdansk, Poland; 3 Department of Human Histology and Embryology, Medical Faculty, Collegium Medicum, University of Warmia and Mazury in Olsztyn, 10-082 Olsztyn, Poland; jolanta.kiewisz@uwm.edu.pl; 4 Central Clinical Laboratory, University Clinical Centre, 80-214 Gdansk, Poland; mdabrowska@uck.gda.pl; 5 The Center of Postgraduate Medical Education, 1st Department of Obstetrics and Gynecology, University of Gdansk, 01-004 Warsaw, Poland; grzegorz.jakiel1@o2.pl; 6 Department of Gamete and Embryo Biology, Institute of Animal Reproduction and Food Research, Polish Academy of Sciences, 10-748 Olsztyn, Poland; i.woclawek-potocka@pan.olsztyn.pl; 7 Laboratory of Recombinant Vaccines, Intercollegiate Faculty of Biotechnology, University of Gdansk, 80-307 Gdansk, Poland; lukasz.rabalski@biotech.ug.edu.pl

**Keywords:** COVID-19, SARS-CoV-2, vaccination, Pfizer/BioNTech, Roche, DiaSorin, Abbott, assay comparison

## Abstract

Background: The introduction of the vaccination against SARS-CoV-2 infection creates the need for precise tools for the quality control of vaccination procedures, detection of poor humoral response, and estimation of the achieved protection against the disease. Thus, the study aimed to compare the results of the anti-SARS-CoV-2 tests to evaluate the application of the WHO standard unitage (the binding antibody units; BAU/mL) for a measurement of response to the vaccination. Methods: Patients undergoing vaccination against SARS-CoV-2 with Pfizer/BioNTech BNT162b2 (BNT162b2) (*n* = 79), referred for SARS-CoV-2 antibody measurement prior to vaccination and 21 days after dose 1, and 8, 14, and 30 days after dose 2 were included. The sera were tested with three assays: Elecsys SARS-CoV-2 S (Roche), LIAISON^®^ SARS-CoV-2 TrimericS IgG (DiaSorin), and SARS-CoV-2 IgG II Quant (Abbott). Results: The three assays showed varying correlations at different time points in the study. The overall agreement for all samples was moderate to high (ρ = 0.663–0.902). We observed the most uniform agreement for the day of dose 2 (ρ = 0.775–0.825), while it was least consistent for day 8 (ρ = −0.131–0.693) and 14 (ρ = −0.247–0.603) after dose 2. The dynamics of changes of the SARS-CoV-2 antibody levels in patients without history of prior SARS-CoV-2 infection appears homogenous based on the Roche results, more heterogenous when considering the DiaSorin results, and in between for the Abbott results. Conclusions: The results highlight the need for further work on the international standard of measurement of SARS-CoV-2 Ig, especially in the era of vaccination. The serological assays can be useful to detect IgG/IgM antibodies to assess the response to the vaccination. However, they cannot be used interchangeably. In terms of the evaluation of the immune response to the BNT162b2 vaccine, Roche and Abbott kits appear to be more useful.

## 1. Introduction

The vaccination against severe acute respiratory syndrome coronavirus 2 (SARS-CoV-2) infection is changing the epidemic situation in countries undertaking mass vaccination programs. Therefore, it is relevant to define and refine vaccination assessment methods to determine the vaccination course and optimize epidemic management protocols.

A variety of kits are used for testing the level of antibodies in blood with ELISA methods. Various viral proteins and different classes of antibodies can be tested. However, the quality of the technology itself and its dependence on multiple variables makes it an appropriate technology for initial measurements for scientific research. For clinical applications, in vitro diagnostic (IVD) kits are better suited as they are simple to perform and, as the systems are automated, the results are not dependent on the skills of the laboratory staff.

The role of antibody testing and the need for continuous evaluation of available tests has been discussed early during the pandemic [1,2] and is still an open issue [3]. To date, the comparative studies of various types of assays measuring levels of anti-SARS-CoV-2 antibodies have mainly relied on test results from COVID-19 patients and recovered patients [4,5,6,7,8,9,10,11,12,13,14,15]. They have primarily addressed qualitative issues such as the presence of antibodies in the blood and the effectiveness of the used tests in detecting infection. Preliminary systematic review and meta-analysis analyzed the usage of the serological tests in patients suspected or known to be infected [16,17]. Serological tests that detect antibodies against the nucleocapsid (N) and the spike (S) protein of SARS-CoV-2 were compared [18,19]. As an example, Trabaud et al. [19], evaluated eight tests. Half of the tests tested total Ig and the others IgG. Four of the tests assessed the binding to the N protein of the virus, while the others tested the binding to the S protein or its receptor-binding domain (RBD) epitope. The reported sensitivity referred to the percentage of positive results in individuals depending on the time of the onset of disease symptoms. Thus, these data were not based on objective and measurable markers of disease. Moreover, this study did not consider asymptomatic individuals, nor had it included an extended follow-up period after contracting COVID-19 [19]. Similarly, the study of Naaber et al. [20] concerned investigating the utility of tests to detect or confirm an immune response in PCR positive patients. A study by Perkmann et al. [21] compared the quality of automated assays. This study of sensitivity, specificity, and quality parameters based on negative samples and 65 SARS-CoV-2 RT-PCR confirmed samples measured 41 days (median time) after symptoms or RT-PCR confirmation. The authors concluded that SARS-CoV-2 antibody tests demanded a very high specificity due to the low seroprevalences of the disease.

Currently available commercial qualitative IVD kits include the LIAISON^®^ SARS-CoV-2 TrimericS IgG (DiaSorin, Stillwater, USA) (DiaSorin) assay, Elecsys Anti-SARS-CoV-2 S (IgG and IgM) (Roche Diagnostics, Mannheim, Germany) (Roche), and SARS-CoV-2 IgG II Quant (IgG) (Abbott, Sligo, Ireland) (Abbott). The DiaSorin assay detects IgG against the trimeric spike glycoprotein of SARS-CoV-2. Roche Ig detection assay is focused on the RBD domain of S1 protein, the same as Abbott (which detects the IgG only). All the assays are easy to use and potentially useful. Recently, they were compared by manufacturers to the WHO International Standard for anti-SARS-CoV-2 immunoglobulin [22]. The intended use of the International Standard is for the calibration and standardization of serological assays detecting antibodies binding and neutralizing SARS-CoV-2 [23].

The primary aim of the current study was to unify the results of the anti-SARS-CoV-2 tests by converting them into the binding antibody units per milliliter (BAU/mL) as defined by the WHO standard and to evaluate their clinical value for confirmation of prior infection history and evaluation of the response to vaccination.

## 2. Materials and Methods

### 2.1. Ethical Policy

Ethical approval was received from the Ethics Committee at the Gdansk Regional Medical Board (No KB-4/21). All participants gave written informed consent for providing blood samples.

### 2.2. Participants

The study included 79 randomly selected participants who underwent SARS-CoV-2 vaccination between 4 January 2021 and 11 March 2021. Persons taking immunosuppressants or allergy medication within one month before vaccination were excluded from the study. Participants were divided into two groups based on their status of the SARS-CoV-2 infection established based on the antibody test performed on the day of the first dose of the vaccine. The patients with a history of SARS-CoV-2 infection (SCV2-positive; *n* = 15; mean age: 37.8 ± 6.13 years (mean ± SD); F/M: 11/4) reported having the infection at least 90 days before vaccination. The SARS-CoV-2 negative patients (SCV2-negative; *n* = 64; mean age: 41.54 ± 11.31 years (mean ± SD); F/M: 55/9) had not reported having contact with a SARS-CoV-2 antigen before vaccination. None of the participants reported any allergic reactions or immune disorders during the vaccination enrollment and medical examination, and all received two 30 ug doses of the Pfizer/BioNTech BNT162b2 mRNA vaccine (Comirnaty; Pfizer/BioNTech) (BNT162b2) with the interval of 21 days between doses. Blood samples were collected from the participants on the day of the first dose prior to vaccination after 21 days (i.e., on the day of the second dose), and 8, 14, and 30 days after the second dose. Samples were handled according to the recommendations of the manufacturer of the tests. 

### 2.3. Assays Characteristics

Three assays were used to determine antibody levels: Elecsys Anti-SARS-CoV-2 S (Roche), LIAISON^®^ SARS-CoV-2 TrimericS IgG assay (DiaSorin), and Architect SARS-CoV-2 IgG II Quant (Abbott). Manufacturer-reported assay characteristics measuring ranges, analytic sensitivity, and detection limits are provided in Table 1. 

The Roche assay detects antibodies (IgG and IgM) to the SARS-CoV-2 spike (S) protein RBD in human serum and plasma. The DiaSorin assay identifies IgG antibodies against the N-terminal S1 (subunit that holds RBD domain that attaches to the angiotensin-converting enzyme 2 (ACE2) receptor) and C-terminal S2 (transmembrane subunit) glycoprotein of the SARS-CoV-2 virus. The Abbott assay detects IgG antibodies to the SARS-CoV-2 S protein RBD in human serum and plasma. All three tests were validated against the WHO International Standard for anti-SARS-CoV-2 immunoglobulin. The number of antibodies in the tested samples was quantified in the units specific for each assay (units per milliliter (U/mL) for Roche and arbitrary units per milliliter (AU/mL for DiaSorin and Abbott) and were converted to BAU/mL according to the manufacturers’ information regarding the WHO standard. The conversion to BAU for Roche, DiaSorin, and Abbott tests were: U/mL × 1.029, AU/mL × 2.6, and AU/mL × 0.142, respectively. The results were analyzed for all the collected samples and separately for the different clinical situations and time points. 

When the upper estimating range was acquired, serum samples were diluted automatically using the reagents supplied by the manufacturer (Roche, limited to 10×), manually with antibody-free serum (DiaSorin, no need for dilution more than 10×), or automatically or manually using the reagents supplied by the manufacturer (Abbott, limited to 2×) and analyzed again. The level of antibodies in the blood of the SCV2-positive group after the first dose of the vaccine exceeded the detection range of the Roche kit and the blood samples from these patients at the post-vaccination time-points were excluded from subsequent comparisons. Thirty-three samples (51.56%) from the SCV2-negative group tested 8 days after the second vaccine dose exceeded the detection range of the Roche kit and thus the results were excluded from further analysis. The results of the remaining 31 patients of the SCV2-negative group were within the detection range of the Roche kit and were used for comparison.

### 2.4. Statistical Analysis

Statistical analyses were performed using R packages (tidyverse, mcr) [24,25,26]. Passing–Bablok regression equations were used to estimate the relationship between the results obtained with different analyses. Bland–Altman plots were used to compare tests graphically to assess bias and check whether the variability in measures was homoscedastic. Correlations among the anti-SARS-CoV-2 immunoglobulin level in-between conducted tests were evaluated by the Spearman rank correlation test. The Wilcoxon signed-rank test was used to assess the differences in mean antibody levels.

## 3. Results

### 3.1. Assay Precision and Accuracy

The precision and accuracy of assays are shown in Table 2. The obtained imprecision rates (I) for compared sets of analytic and evaluated Ig values were satisfactory and did not exceed 4.5%.

### 3.2. Detection of SARS-CoV-2 Antibody within the Whole Study Group

Passing–Bablok regressions and Bland–Altman plots comparing Ig values of the samples included in the study (*n* = 172), collected before the first dose of the vaccine (*n* = 15) and 21 days after the first dose (*n* = 64), and 8 (*n* = 31), 14 (*n* = 31), and 30 (*n* = 31) days after the second dose, measured with Roche, DiaSorin, and Abbott tests, are presented in the Supplementary Materials (Figure S1). The Passing–Bablok linear regression shows correlation between the assays (Roche/DiaSorin (R/D), Roche/Abbott (R/A), and DiaSorin/Abbott (D/A)) (r = 0.684; *p* < 0.0001, r = 0.902; *p* < 0.0001, and r = 0.663; *p* < 0.0001, respectively). A disparity was apparent in many results. The Bland–Altman plots show that as the mean value of Ig levels increases, the differences between the two values become larger (Supplementary Materials Figure S1).

### 3.3. Detection of SARS-CoV-2 Antibody within the SCV2-Positive Group

For the SCV2-positive group, only samples collected before vaccination are included (*n* = 15). The results in this group for all the time points following vaccination were outside the detection limit for the Roche kit and were not included in any comparisons. In the SCV2-positive group of patients who suffered from COVID-19 approximately three to six months prior to vaccination, the correlation between the pairs of tests was as follows: R/D − ρ = 0.514, *p* = 0.050; R/A − ρ = 0.554, *p* = 0.032; and D/A − ρ = 0.821, *p* < 0.0001.

The Passing–Bablok regressions and Bland–Altman plots comparing the same samples tested with Roche, DiaSorin, and Abbott tests for SCV2-positive are presented in Supplementary Materials Figure S2.

### 3.4. Detection of SARS-CoV-2 Antibody within SCV2-Negative Group Prior to Second Vaccine Dose

The SARS-CoV-2 antibody levels in the samples from the SCV2-negative group collected on the day of the second vaccine dose (*n* = 64) showed a high correlation between the compared results of the same samples measured with different diagnostic tests: R/D–ρ = 0.775; *p* < 0.001; R/A–ρ = 0.817; *p* < 0.001; and D/A–ρ = 0.825; *p* < 0.001.

Passing–Bablok and Bland–Altman plots for these samples from the SCV2-negative group evaluated with Roche, DiaSorin, and Abbott tests are presented in Supplementary Materials Figure S3.

### 3.5. Detection of SARS-CoV-2 Antibody within SCV2-Negative Group on Day 8, 14, and 30 after the Second Dose

Following the second vaccine dose, all participants had their SARS-CoV-2 antibody levels measured on days 8, 14, and 30 after the second dose. Only samples from the participants whose results remained within the Roche assay detection limit were included in further comparisons of the results obtained on days 8 (*n* = 31), 14 (*n* = 31), and 30 (*n* = 31). Supplementary Materials Figures S4–S6 show Passing–Bablok and Bland–Altman plots for those three time points. 

Samples tested 8 (Figure S4) and 14 (Figure S5) days after the injection of the second dose of the vaccine presented a very low correlation of test results for R/D and R/A pairs and moderate for D/A (day 8: R/D–ρ = 0.062, *p* = 0.739; R/A–ρ = 0.693, *p* < 0.0001; and D/A–ρ = −1.131, *p* = 0.481; and day 14: R/D–ρ = 0.154, *p* = 0.409; R/A–ρ = 0.603, *p* < 0.0001; and D/A–ρ = −0.247, *p* = 0.180).

The correlation of results of SARS-CoV-2 Ig levels was low to moderate for samples taken 30 days after the second vaccine dose (Figure S6: R/D–ρ = 0.506, *p* = 0.004; R/A–ρ = 0.314, *p* = 0.086; and D/A–ρ = 0.624, *p* = 0.0002). All correlations for each pair of tests and all time points are shown in Table 3.

**Figure 1:**
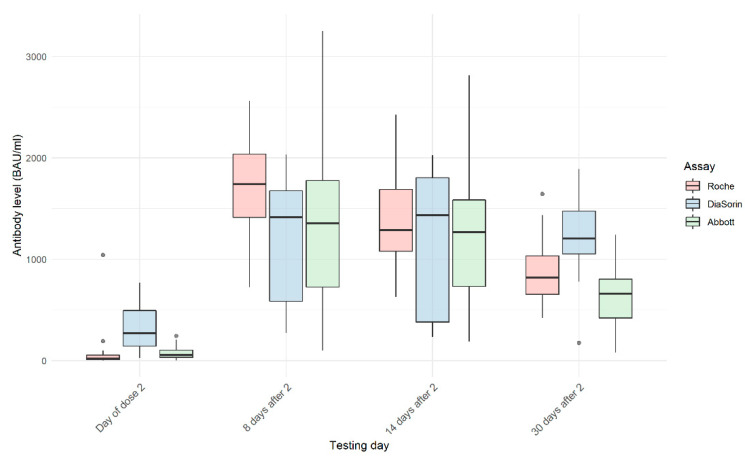
Dynamics of change in SARS-CoV-2 antibody levels in the subset of the SCV2-negative group with four measurements: on the day of the second dose of the vaccine and 8, 14, and 30 days after the second dose. In the SCV2-negative group, all test results were negative on the day of the first dose, therefore they were excluded from the graph.

The dynamics of changes in the antibody levels in tested samples measured with Roche, DiaSorin, and Abbott tests are shown in Figure 1. All three tests show a large significant increase in the mean value of the antibody levels from the day of the second dose of the vaccine to day 8 after the second dose. However, differences between results obtained on day 8 and day 14, and day 14 and day 30 were statistically significant for both comparisons only for the Roche assay (day 8–14: 1665.2 BAU/mL–1349.2 BAU/mL, *p* < 0.0001; and day 14–30: 1349.2 BAU/mL–854.1 BAU/mL, *p* < 0.0001). Mean values of the DiaSorin results in both cases were not statistically significant (day 8–14: 1248.0 BAU/mL–1196.4 BAU/mL, *p* = 0.72; and day 14–30: 1196.4 BAU/mL–1221.0 BAU/mL, *p* = 0.85). In the case of the Abbott test, the difference between day 8 and day 14 was not statistically significant (day 8–14: 1409.5 BAU/mL–1276.5 BAU/mL, *p* = 0.09), while the comparison of day 14 with day 30 showed a significant difference (day 14–30: 1276.5 BAU/mL–631.6 BAU/mL, *p* < 0.0001).

**Figure 2:**
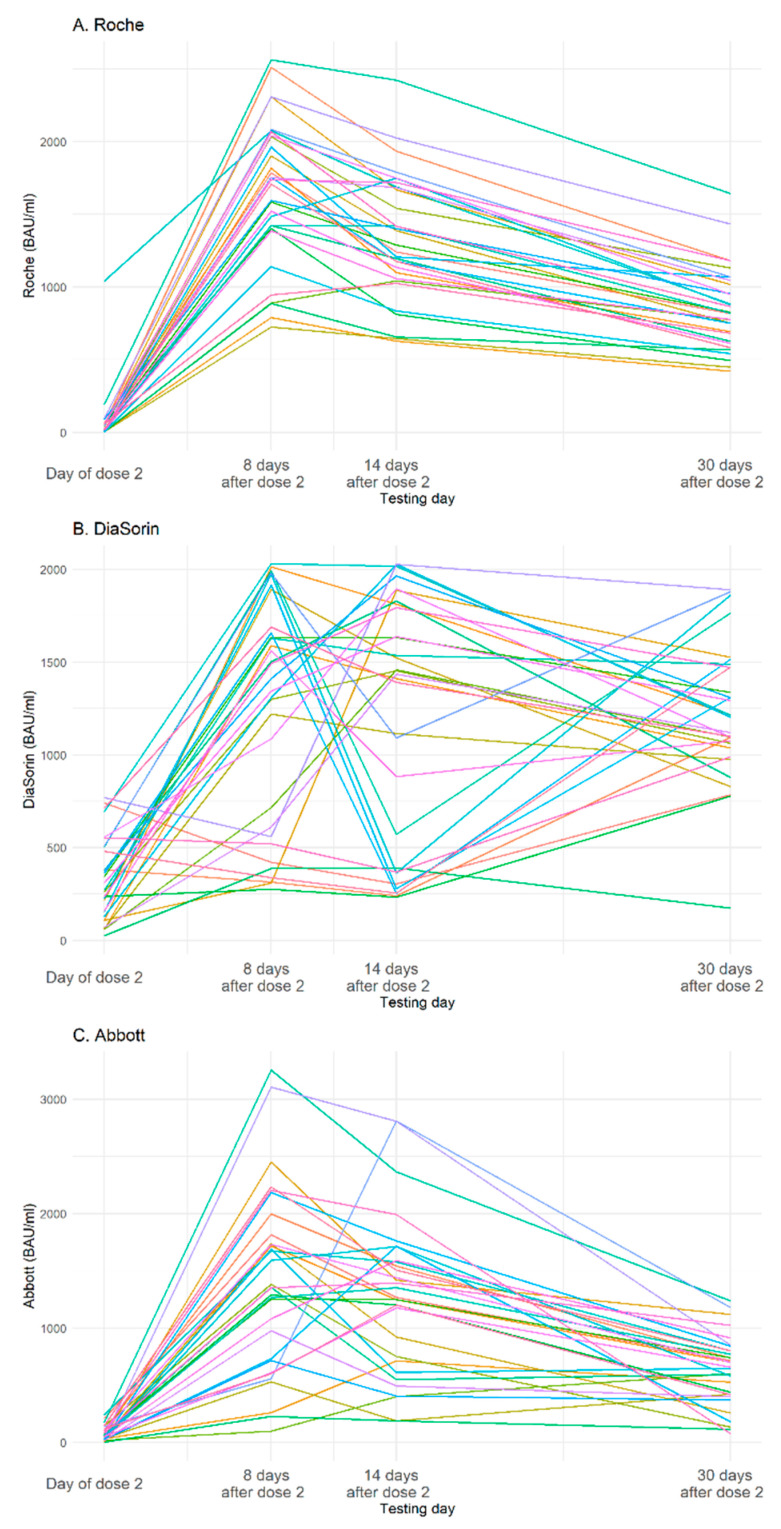
Dynamics of SARS-CoV-2 antibody level changes for individual participants (subset of the SCV2-negative group with four samples per participant) as measured by Roche (**A**), DiaSorin (**B**), and Abbott (**C**) assays. In the SCV2-negative group, all test results were negative on the day of the first dose, therefore they were excluded from the graph.

Figure 2 presents the dynamics of changes of the SARS-CoV-2 antibody levels in individuals in the SCV2-negative subgroup whose Roche results remained within the detection limit at all time points. The profile of the SARS-CoV-2 antibody levels in samples measured with the Roche test shows, as expected, a rise in the antibody levels 8 days after the second dose and a steady decline 14 and 30 days after vaccination. The measurement of the SARS-CoV-2 antibody levels with the DiaSorin test shows diversified profiles and heterogeneous dynamics of the antibodies’ changes in the tested samples. Abbott results are less homogenous than Roche but more consistent than DiaSorin.

## 4. Discussion

The prevalence and high mortality rate associated with SARS-CoV-2 has changed the epidemic, economic, medical, psychological, and sociological situation of entire populations. Available vaccines are based on different technologies and will vary concerning the generated immune response. It is important to validate available methods that can be used to detect previously infected individuals and analyze the response to vaccines. Thus, we examined three IVD kits from analytical solution providers (Roche, DiaSorin, and Abbott kits). We confirmed the high precision and accuracy of the assays as shown in Table 2. However, we did observe an increasing difference between each pair of values when the mean of Ig levels was growing. After division into the SCV2-positive group (confirmed infection approximately three to six months prior to vaccination) and SCV2-negative group, we observed that for the SCV2-positive group, the only results that remained within the detection limit for the Roche kit were those before the first dose of the vaccine. R/D and R/A correlations were moderate and D/A was high. In the SCV2-negative group, all patients had a result within the Roche detection limit only for samples collected on the day of the second vaccine dose. We found high correlations between the Roche, DiaSorin, and Abbott results for samples collected on that day. When analyzing results for SCV-negative patients whose antibody levels remained within the Roche detection limit for all time points after the second vaccine dose, the correlations for R/D and D/A dropped down to negligible levels for days 8 and 14, and rose to a medium level for day 30 after the second dose of the vaccine. The opposite change has been observed for R/A with a moderate correlation on days 8 and 14, and low on day 30. The dynamics of changes of the SARS-CoV-2 antibody levels in SCV2-negative group appear homogenous based on the Roche results, heterogenous when considering the DiaSorin results, and in between for Abbott results.

The Roche, DiaSorin, and Abbott kits tested are of very high quality, require small sample volumes, and have short testing times. All kits appear to be very useful in assessing the humoral response of vaccinated individuals. However, the Roche kit makes response assessment difficult due to calibration at a relatively low level and technically unfeasible sample dilution above 10× (dilution over 10× leads to unreliable results, i.e., unpublishable data). The DiaSorin and Abbott kits have a wide measurement range and practically every antibody level produced in the vaccinated person falls within the scale that can be determined. What is surprising concerns the large variability in antibody levels when assessing the dynamics of their changes in individual patients, contradicting our knowledge and the results of the Roche kit. 

Previously published studies that compared various SARS-CoV-2 antibody assays have mainly focused on qualitative issues and relied on samples from COVID-19 patients. The continuous evolution of the assays also makes the comparisons more difficult. For example, the study by Perkmann et al. [21] evaluated previous versions of the Roche (Anti-SARS-CoV-2 (against nucleocapsid), not Anti-SARS-CoV-2 S assay), DiaSorin (SARS-CoV-2 S1/S2 IgG, which has been replaced by SARS-CoV-2 TrimericS IgG test), and Abbott (SARS-CoV-2 IgG (against nucleocapsid), not SARS-CoV-2 IgG II Quant) assays. All the assays have had the test domains changed to allow for a better ability to measure the quantitative response to vaccination. The test antigen in the DiaSorin kit (recombinant trimeric spike glycoprotein) now allows for the measurement of the level of antibodies against different epitopes of the S protein. The Roche and Abbott kits are focused on the detection of the antibodies against the receptor-binding domain (RBD) of the spike (S) protein (IgG+IgM and IgG, respectively). The BNT162b2, Moderna, and AstraZeneca vaccines elicit antibodies to the RBD of the spike protein. Thus, this facilitates a more precise evaluation of the response to these specific vaccines but may make it more difficult to evaluate vaccines with a broader spectrum of induced responses. 

To date, few studies evaluated the immunoassays in terms of the immune response to vaccination. Some initial studies used only the ELISA method [27]. A publication on a similar topic to our study includes another paper by Perkmann et al. [28] who compared five tests (including three that were evaluated in our study) using sera from 69 individuals who did not suffer from SARS-CoV-2 infection. The samples used were obtained 21 days after the first dose of the BNT162b2 vaccine. The correlations between the pairs of tests that overlap with our study obtained by Perkmann are similar to those obtained by the current study (R/D: ρ = 0.775 vs. ρ = 0.83; R/A: ρ = 0.817 vs. ρ = 0.88; and D/A: ρ = 0.825 vs. ρ = 0.90). Both studies involved a comparable group of subjects (64 vs. 69 subjects) of similar age (41.6 vs. 42 years) and samples were taken exactly on the same day after the first dose of the same vaccine. This is, however, the only time point where all three pairs of tests showed high correlations (Table 3). 

We have analyzed the antibody levels at specified time points after the second dose of the vaccine. Our goal was to assess if the weak correlation is caused by the IgM that emerges after the first contact with the antigen and persists for approximately six weeks [29]. However, our results from samples obtained at time points that should have either no IgM (SVC2-positive prior to vaccination) or minimal IgM (SVC2-negative 30 days after the second vaccine dose, i.e., 7 weeks after the first dose) showed only a medium correlation between the tests’ results. Simulataneously, we have seen the lowest correlation in the results from days 8 and 14 after the second vaccine dose. These results could be explained by the presence of IgM measured by the Roche assay but not by DiaSorin and Abbott assay.

This study indicates a further need for unification of the standard and cooperation between assay manufacturers to agree on the cross-utility of their tests. Despite manufacturers’ claims about the value of the tests, they require external validation to detect problems that may be difficult to define within a commercial organization. Moreover, assays must be validated for virus neutralization to assess their usefulness both in the measurement of SARS-CoV-2 Ig levels and confirmation of SARS-CoV-2 infection.

In all three cases, the companies confirmed that the results for the WHO reference panel samples are as expected and are easily converted to units of BAU/mL of the WHO International Standard. The intended use of the WHO International Standard is for the calibration and standardization of serological assays detecting anti-SARS-CoV-2 neutralizing antibodies. The results obtained do not show any meaningful correlation between the tested kits, making it impossible to cross-evaluate the results between the assays.

The WHO standard is based on pooled lyophilized human plasma from convalescent patients. According to the instructions for its use, it ”can be used to assist the comparison of assays detecting the same class of immunoglobulins with the same specificity (e.g., anti-receptor- binding domain IgG, anti-N IgM, etc.)” [23]. However, the use of a standard for binding assays based on a mixture of polyclonal antibodies that arose naturally in different convalescents will always lead to the type of observation we have presented even for the same class of immunoglobulins with the same specificity. To achieve standardization of the kits, their manufacturers would have to be obliged to use the same proteins that bind to the tested antibodies. The DiaSorin assay uses the recombinant trimeric SARS-CoV-2 spike protein, whereas the Roche and Abbott assays are based on a recombinant element of this complex, the receptor-binding domain (RBD). However, even in the case of these two manufacturers, they used a different method of combining the detected antibodies with the detection system: Roche uses the same RBD (labelled with the ruthenium complex), whereas Abbott uses a classical technique based on monoclonal antibodies against human IgG, labelled in their system with acridine derivatives. Hence, the Roche kit detects any Ig against RBD, while the Abbott kit detects only the IgG fraction. However, even this does not explain such an inconsistent correlation between the assays. The use of the BAU unitage introduced by the standard is being adopted by the assay manufactures and recommended to be used for any studies and trials [30], becoming a simple tool used by the public.

We are aware that empirical results presented herein should be considered in light of methodological and substantive limitations that require consideration. The outcomes were based on a relatively small group of patients. Thus, there is a need to conduct an experiment that comprises study groups evaluated for the humoral response following vaccination with BNT162b2 and other vaccines. Moreover, validation should be performed with the independent neutralization antibody measurements. However, due to relatively sparse knowledge not only about SARS-CoV-2 but also other coronaviruses, we observe methodological deficiencies in this field. Antibody levels sufficient to neutralize the virus and block its entry into the host cell, in addition to multiplication, have not been yet determined. Assay manufacturers report correlation of serum neutralization to the antibody levels only in terms of the agreement with the positive/negative determination of the presence of antibodies. As the research of SARS-CoV-2 has evolved in the last months, there are limited data on post-infectional duration and biological and genetic aspects influencing acquired immunity. The short duration of acquired immunity to coronaviruses [31] or lack of immunological response can affect the number of detected antibodies. Moreover, the response to the SARS-CoV-2 infection is also antibody class-dependent. The number of SARS-CoV-2 virus-specific IgM, IgA, and IgG classes in blood changes depending on the number of days after the onset of the signs of infection [32,33,34]. It is of particular importance and can affect the assay results as all of the tests that were utilized recognize IgG class and only the Roche assay recognizes IgG and IgM simultaneously. The antibody titers can also change depending on age or gender. Thus, as the immune system goes through progressive biological changes, it is also relevant to consider immune-modulating agents when organizing a study group [35].

## 5. Conclusions

In conclusion, we found varying correlations between all tested assays and that the WHO International Standard has not improved the standardization of serological assays detecting anti-SARS-CoV-2 binding antibodies. However, we have demonstrated that all assays can be used clinically for the identification of infected subjects, the convalescents’ Ig levels, and the confirmation of the individual vaccination response. In our opinion, greater precision was exhibited by the Roche and Abbott kits. The DiaSorin and Abbott tests show more extensive scopes of estimations, which reduces the need for sample dilution. In contrast, the Roche results are better concerning the detection of a humoral response to vaccination. It also needs to be noted that as the studied assays measure only the humoral response to vaccination, no conclusion can be reached about cellular immunity and overall protection against SARS-CoV-2. Moreover, more research is needed to ascertain the correlation of the antibody levels measured with a commercial assay with neutralization to have a better insight into immunogenicity and efficacy of vaccines.

As the values generated by all assays were markedly different even after conversion to WHO International Standard units (that may be assay-specific due to the recognition of varied antibody classes and different epitopes), assay-specific and personalized interpretation is required. To incorporate the marked differences in the scale observed when assay results are converted to BAU, we would recommend using an additional identifier together with the common unit (e.g., BAU_RBD-IgG_/mL). The use of identifiers would clarify the interpretation of the conducted analysis, allow for the accurate interpretation of the obtained results, and limit the risk of making direct comparisons between test results from assays based on different immunoglobulins and epitopes. Such modification would bring us closer to solving the pressing problem regarding the objective assessment of the immune response of the vaccinees.

## Figures and Tables

**Table 1:** Characteristics of the assays.

Characteristics	Elecsys Anti-SARS-CoV-2 S	LIAISON^®^ SARS-CoV-2 TrimericS IgG	SARS-CoV-2 IgG II Quant
**Specified by manufacturer:**			
Assay type	Automated		
Dilution method	Automated, 10×	Manual, 10×	Automated, 2×
Testing time	18 min	35 min	29 min
Test principle	Double-antigen sandwich principle. Electrochemiluminescence detection (ECLIA)	Indirect immunoassay. Chemiluminescence detection (CLIA)	Chemiluminescence microparticle immunoassay (CMIA)
Calibration	2 points		6 points
Calibrators frequency	Started with new reagent lot/quality control findings outside the defined limits		
Traceability	Standardized against the internal Roche standard for anti-SARS-CoV-2 S/from date 12.01.2021. Standardized against the WHO IS: NIBSC 20-136	Correlation with Microneutralization Test (MNT)/from date 02.02.2021. Standardized against the WHO IS: NIBSC 20-136	Standardized against the internal Abbott standard for anti-SARS-CoV-2 S/correlation with Microneutralization Test (MNT)/from date 02.2021. Standardized against the WHO IS: NIBSC 20-136
Sample material	Serum		
Sample volume	12 uL	10 uL	25 uL
Limit of detection (LoD)	0.35 U/mL	0.712 AU/mL	6.8 AU/mL
Limit of quantification (LoQ)	0.40 U/mL	1.63 AU/mL	21.0 AU/mL
Measuring range	0.40–250 U/mL	1.85–800 AU/mL	21.0–40,000.0 AU/mL
BAU conversion	U/mL × 1.029	AU/mL × 2.6	AU/mL × 0.142
**Laboratory specific:**			
Controls frequency	Daily		

**Table 2:** Precision and accuracy of the assays.

Control Sample	Roche (U/mL)		DiaSorin (AU/mL)		Abbott (AU/mL)		
	PreciControl Anti-SARS-CoV-2 S p1	PreciControl Anti-SARS-CoV-2 S p2	SARS-CoV-2 TG Control Set p1	SARS-CoV-2 TG Control Set p2	SARS-CoV-2 IgG II Quant Control	SARS-CoV-2 IgG II Quant Control +1	SARS-CoV-2 IgG II Quant Control +2
LOT	526346	526347	311031	212031	23268FN00		
Nominal value	n/a	8.41	n/a	37.5	2.3	166	602.5
Range	0.000–0.399	5.887–10.933	0.0–6.0	26.38–48.8	0.0–18.0	91.3–240.7	331.4–873.6
Average	<0.400	7.86	<1.85	38.29	3.075	172.5	650.84
SD	n/a	0.193	n/a	1.323	n/a	5.81	14.47
I (%)	n/a	2.5	n/a	3.46	n/a	4.4	3.3
B (%)	n/a	−6.6	n/a	2.1	n/a	3.9	8.0

Abbreviations: SD = standard deviation; I = imprecision; and B = bias.

**Table 3:** Correlations between each pair of tests for Roche, DiaSorin, and Abbott for all samples and each subgroup.

	All Samples	SCV2-Positive	SCV2-Negative			
		Before Dose 1	Before Dose 2	8 d after Dose 2	14 d after Dose 2	30 d after Dose 2
■ **Pair of tests**	ρ	ρ	ρ	ρ	ρ	ρ
■ Roche ■ /DiaSorin	0.684 *	0.514 *	0.775 *	0.062	0.154	0.506 *
■ Roche ■ /Abbott	0.902 *	0.554 *	0.817 *	0.693 *	0.603 *	0.314
■ DiaSorin ■ /Abbott	0.663 *	0.821 *	0.825 *	−0.131	−0.247	0.624 *

* statistically significant; strength of correlation highlighted with color.

## Data Availability

The data presented in this study are available on request from the corresponding author.

## Supplementary Materials

The following are available online at https://www.mdpi.com/article/10.3390/vaccines9080840/s1. Figure S1: Passing-Bablok and Bland-Altman plots for the same samples compared with Roche, DiaSorin and Abbott tests, for the all tested samples at all time points (*n* = 172); Figure S2: Passing-Bablok and Bland-Altman plots samples from SCV2-positive collected before the first dose of the vaccine (*n* = 15), compared with Roche, DiaSorin and Abbott tests; Figure S3: Passing-Bablok and Bland-Altman plots for SARS-CoV-2 antibody level in the SCV2-negative group on the day of the second vaccine dose; Figure S4: Passing-Bablok and Bland-Altman plots for the subgroup of the SCV2-negative group that remained within the detection range for the time points after the second dose of the vaccine—8 days after dose 2 (*n* = 31); Figure S5: Passing-Bablok and Bland-Altman plots for the subgroup of the SCV2-negative group that remained within the detection range for the time points after the second dose of the vaccine—14 days after dose 2 (*n* = 31); Figure S6: Passing-Bablok and Bland-Altman plots for the subgroup of the SCV2-negative group that remained within the detection range for the time points after the second dose of the vaccine—30 days after dose 2 (*n* = 31).
