# Supplementary material for: Usefulness of IVD Kits for the Assessment of SARS-CoV-2 Antibodies to Evaluate the Humoral Response to Vaccination
Source: Vaccines (Basel). 2021 Jul 31;9(8):840. doi: 10.3390/vaccines9080840 (PMC8402409; doi:10.3390/vaccines9080840)
Supplement: Supplementary file 1 [file vaccines-09-00840-s001.zip › vaccines-1249682-supplementary.pdf]

vaccines

MDPI

Supplementary Materials

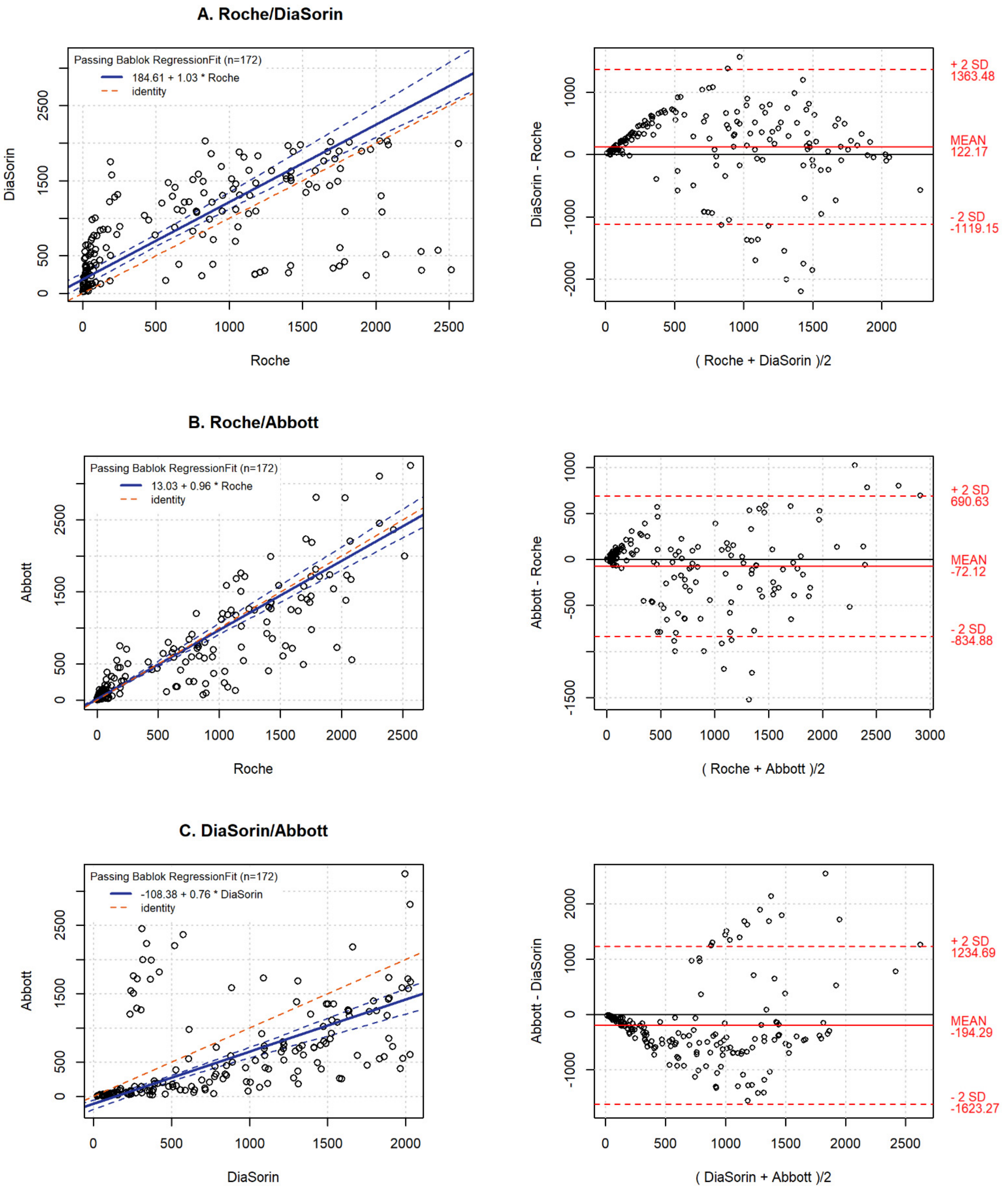

Figure S1. Passing-Bablok and Bland-Altman plots for the same samples compared with Roche, DiaSorin and Abbott tests, for the all tested samples at all time points (n = 172).

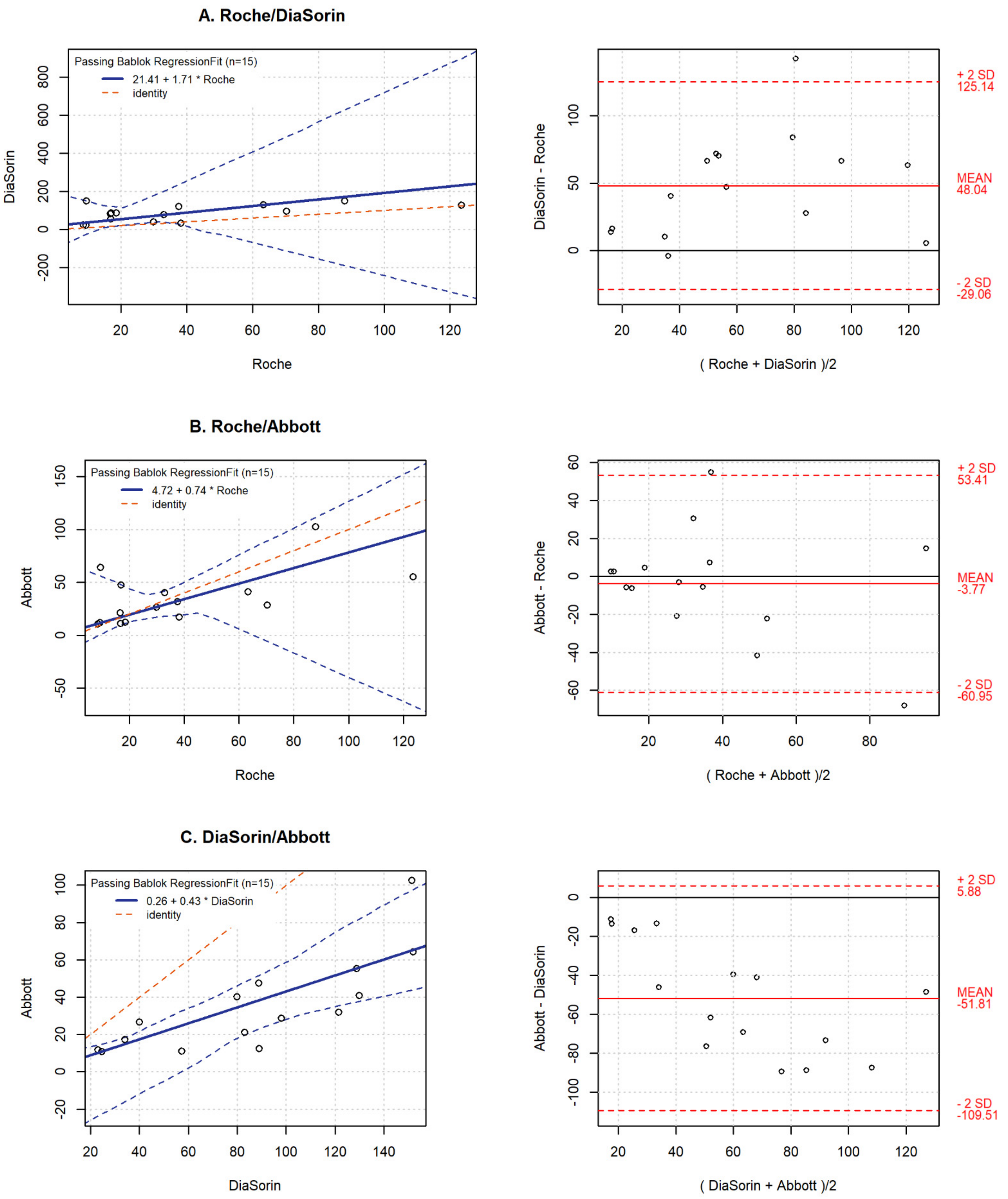

Figure S2. Passing-Bablok and Bland-Altman plots samples from SCV2-positive collected before the first dose of the vaccine (n = 15), compared with Roche, DiaSorin and Abbott tests.

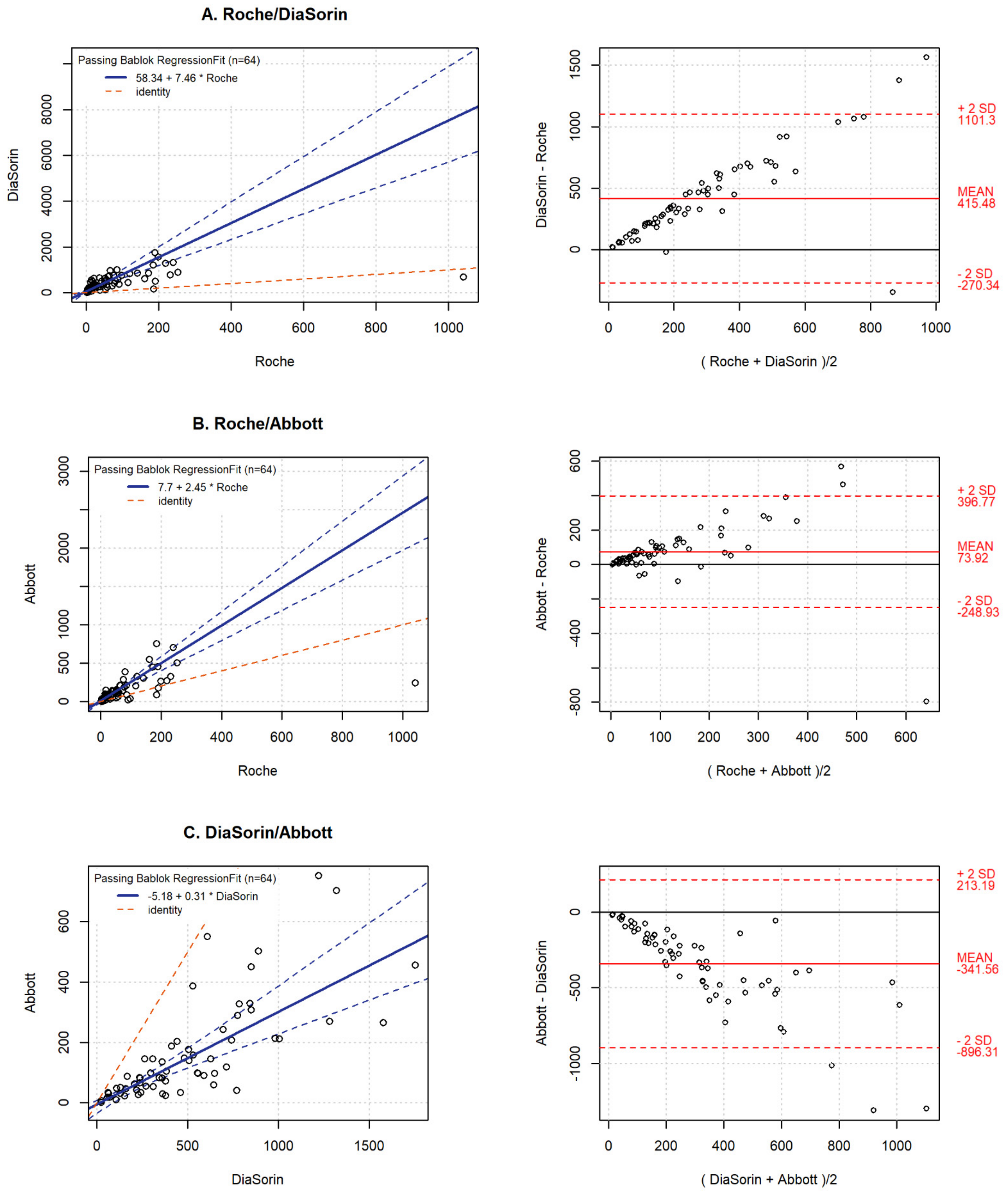

Figure S3. Passing-Bablok and Bland-Altman plots for SARS-CoV-2 antibody level in the SCV2-negative group on the day of the second vaccine dose.

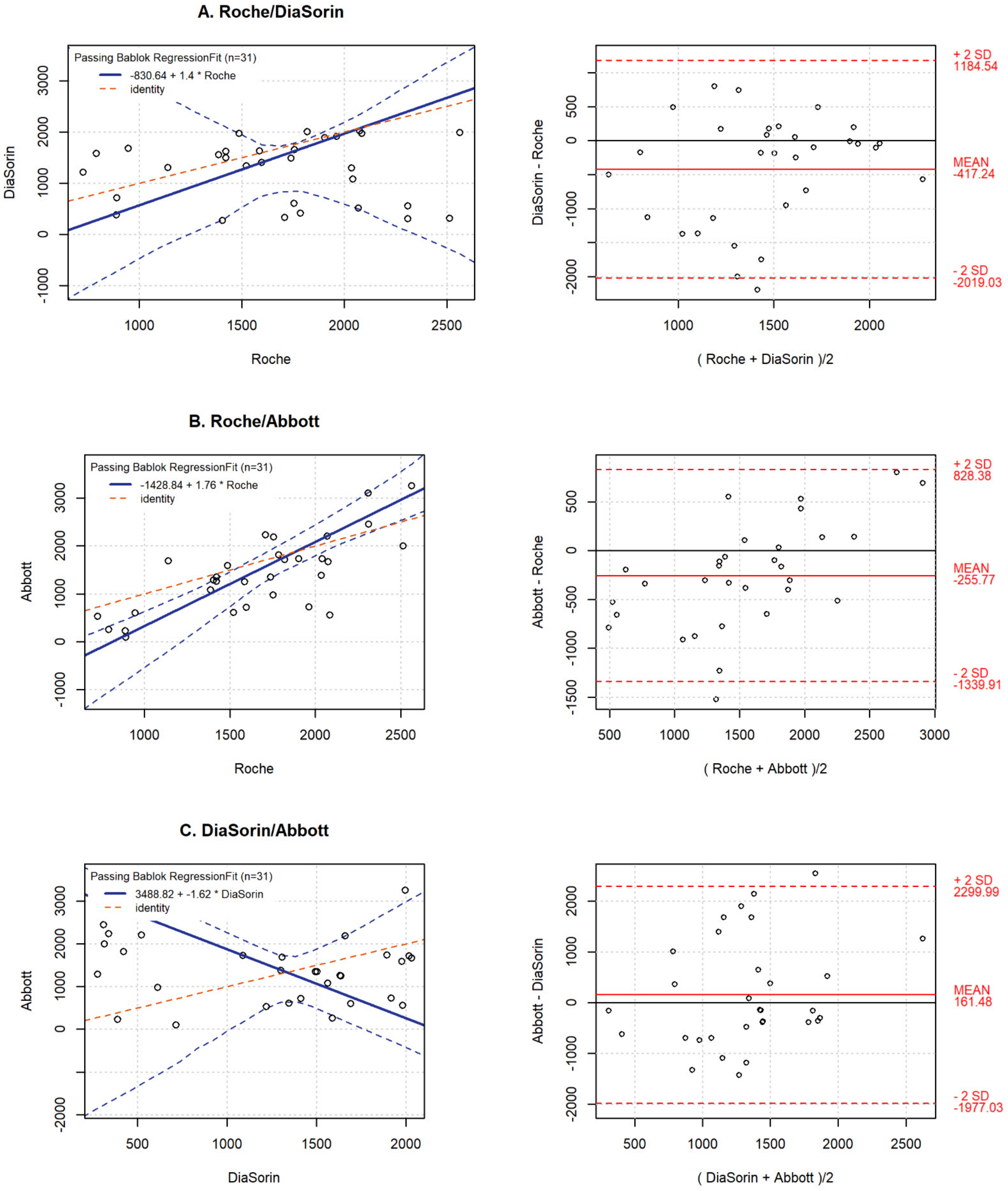

Figure S4. Passing-Bablok and Bland-Altman plots for the subgroup of the SCV2-negative group that remained within the detection range for the time points after the second dose of the vaccine – 8 days after dose 2 (n = 31).

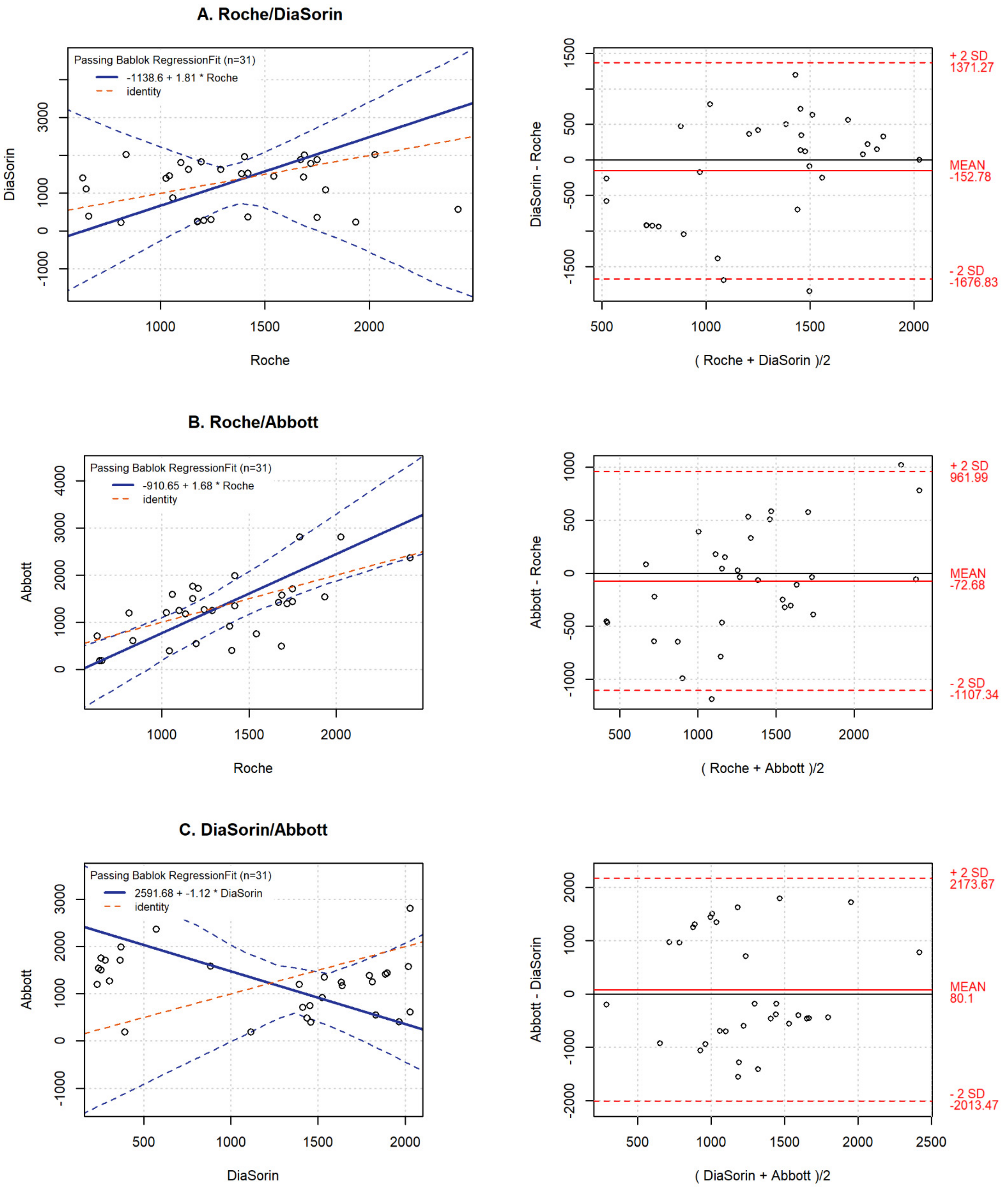

Figure S5. Passing-Bablok and Bland-Altman plots for the subgroup of the SCV2-negative group that remained within the detection range for the time points after the second dose of the vaccine – 14 days after dose 2 (n = 31).

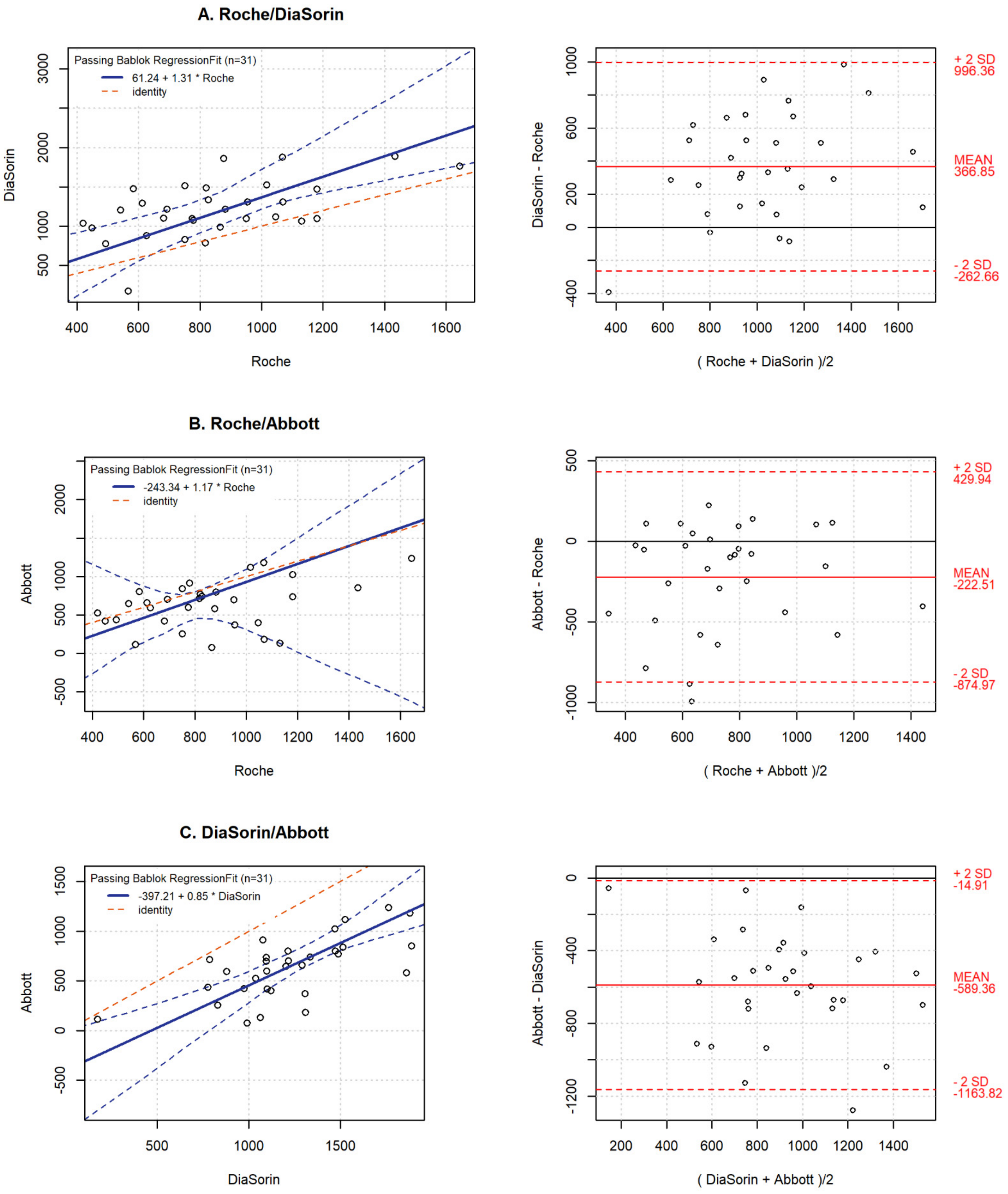

Figure S6. Passing-Bablok and Bland-Altman plots for the subgroup of the SCV2-negative group that remained within the detection range for the time points after the second dose of the vaccine – 30 days after dose 2 (n = 31).
